# Prevalence of Decisional Regret and Associated Risk Factors in Patients With Hepatitis B Undergoing Pegylated Interferon Alpha Therapy

**DOI:** 10.1155/grp/5837940

**Published:** 2026-10-26

**Authors:** Yufen Lin, Huan Xiong, Shuanglian Yi, Shanshan Xie

**Affiliations:** ^1^ Department of Gastroenterology The National Key Clinical Specialty Zhongshan Hospital of Xiamen University School of Medicine Xiamen University Xiamen Fujian Province China xmu.edu.cn; ^2^ Clinical Research Center for Gut Microbiota and Digestive Diseases of Fujian Province Xiamen Key Laboratory of Intestinal Microbiome and Human Health Xiamen Fujian Province China; ^3^ Department of Digestive Disease Institute for Microbial Ecology School of Medicine Xiamen University Xiamen Fujian Province China xmu.edu.cn

**Keywords:** antiviral therapy, decisional regret, hepatitis B, pegylated interferon alpha

## Abstract

**Background:**

Pegylated interferon alpha has been shown to enhance the likelihood of achieving a functional cure in patients with hepatitis B. However, it was associated with a greater number of contraindications and adverse reactions compared with nucleotide analogs. Current research has placed comparatively less emphasis on the potential for this treatment to induce decision regret among patients.

**Aim:**

The aim of this study was to investigate the current status of decision regret in hepatitis B patients undergoing interferon therapy and to explore its influencing factors.

**Method:**

A cross‐sectional study was conducted in a comprehensive hospital in Xiamen, Fujian Province, China, from February 20 to June 13, 2024. The study recruited hepatitis B patients undergoing treatment with pegylated interferon alpha. The survey encompassed general information, the All Aspects of Health Literacy Scale, the Perceived Social Support Scale, the Control Preferences Scale, the Decisional Conflict Scale, and the Decision Regret Scale.

**Results:**

A total of 130 questionnaires were analyzed. Only 13.85% of patients had no regrets at all about the decision, 34.60% experienced mild regret, and the proportion of patients with moderate to severe regrets reached 51.50%. Mild impact on life, active decision‐making roles, passive decision‐making roles, and decision conflict were the influencing factors of decisional regret in hepatitis B patients on pegylated interferon alpha therapy, which together explained 29.6% of the total variation.

**Conclusion:**

Hepatitis B patients undergoing pegylated interferon alpha therapy had a high percentage of decisional regret. Healthcare professionals should pay close attention to this phenomenon. In the future, they can investigate additional potential influencing factors and promptly develop intervention strategies to effectively reduce decision regret.

## 1. Introduction

Hepatitis B virus (HBV) infection is a significant public health issue. In 2019, the World Health Organization (WHO) estimated that 296 million people were chronically infected with hepatitis B, leading to 820,000 deaths from chronic liver disease, primarily due to cirrhosis and liver cancer [1]. The WHO is aimed at eliminating viral hepatitis as a public health threat by 2030, with targets including a 90% reduction in new chronic hepatitis B infections, a 65% decrease in mortality, a 90% diagnosis rate, and an 80% treatment rate by that year [2]. Although some countries have made progress in reducing seroprevalence in children under 5 through vaccination, few have met the goals for mortality and treatment rates [3]. It is crucial to enhance support for hepatitis B treatment interventions strategically to achieve elimination promptly.

The clinical goals of treatment for HBV‐infected patients are to maximize long‐term inhibition of HBV replication, delay and reduce the occurrence of hepatic failure, cirrhosis, and other complications, and improve the quality of life of patients [4]. For some eligible patients, a functional cure, that is, sustained (≥ 24 weeks posttreatment) decline in HBsAg with or without seroconversion to hepatitis B surface antibodies (anti‐HBs) and undetectable HBeAg and serum HBV DNA after a limited course of treatment, should be pursued [5]. The main antiviral drugs currently used for the treatment of HBV infection are nucleotide analogs (NAs) and pegylated interferon alpha (Peg‐IFN‐*α*). Studies have shown that chronic hepatitis B patients receiving NAs antiviral therapy have a 10‐year cumulative HBsAg clearance rate of only 2.11%, making it difficult to achieve a functional cure [6]. Peg‐IFN‐*α* add‐on NAs, or NAs switched to Peg‐IFN‐*α* could significantly improve HBsAg clearance [7]. However, there are more contraindications to the use of Peg‐IFN‐*α* than NAs, and Peg‐IFN‐*α* has been used in less than 5% of patients in the real world [8]. Furthermore, there may be a variety of adverse effects such as influenza‐like syndrome, myelosuppression, alopecia, and insomnia, as well as a relatively high economic burden [9]. Although achieving a functional cure is the main driver of patients′ therapeutic decisions and adverse reactions of Peg‐IFN‐*α* are tolerated by most patients, it is worth noting that patients preferred tablets as the preferred route of administration, and adverse effects remain an important reason for patients to refuse and discontinue Peg‐IFN‐*α* [10]. The symptomatic experience of adverse reactions and the impact of adverse reactions on baseline functional status and daily life may lead to a negative cognitive‐emotional phenomenon in patients with past health decisions, which is called decisional regret [11].

Decision regret is increasingly being used as a patient‐reported outcome, often as an indicator of the quality of the decision‐making process between clinicians and patients [12]. Higher levels of decisional regret are associated with poor adherence, poorer health status, impaired quality of life, and a high risk of anxiety and depression [13–15]. Previous studies have shown that sociodemographic factors (e.g., age, education level, and social support), health literacy, decision conflict, and decision participation may affect the degree of decision regret [13, 16]. Currently, there is a lack of data related to patients′ decisional regrets about Peg‐IFN‐*α* treatment. Therefore, the aim of this study was to investigate the prevalence of decision regret in interferon therapy for patients with hepatitis B, as well as to identify possible factors contributing to regret, in order to provide a reference for improving satisfaction with decision‐making regarding antiviral therapy for hepatitis B patients.

## 2. Methods

### 2.1. Design and Procedure

A cross‐sectional survey was conducted from February 20, 2024, to June 13, 2024, at a comprehensive Grade III Class A hospital—the highest level in China′s hospital classification system, representing the most advanced comprehensive hospital capabilities in the region—in Xiamen, Fujian Province. Inclusion criteria were as follows: patients with chronic HBV infection, HBsAg positive for 6 months or more, age ≥ 18 years, and patients on interferon therapy. Exclusion criteria were as follows: coinfection with hepatitis A, C, D, or human immunodeficiency virus; decompensated stage cirrhosis; hepatocellular carcinoma or any other tumor; severe infections; underlying diseases such as retinal disease, heart failure, and chronic obstructive pulmonary disease; pregnant or lactating women; history of psychiatric disorders; uncontrolled epilepsy; autoimmune disorders; thyroid disorders; diabetes mellitus; hypertension; and heart disease [17]. Survey invitations were sent to those who met the inclusion and exclusion criteria. After obtaining informed consent, paper questionnaires or electronic questionnaire QR codes were distributed to the participants according to their preferences. Survey team members were trained to explain the purpose of the survey and how to fill it out to participants using a standardized guide. Paper questionnaires were required to be collected on the spot, checked for omissions and errors, and corrected with the participants. Missing questionnaires that could not be completed were eliminated. The electronic questionnaire was set as mandatory for all questions in order to avoid omissions. Background settings were adjusted to allow the same device to answer only once to prevent repeated responses. The study obtained ethical approval from Hospital′s Medical Ethics Committee under Reference Number xmzsyyky2024‐031.

### 2.2. Measurements

#### 2.2.1. General Information

The general information in this study included gender, age, marital status, fertility status, education level, employment status, distance to hospital, perceived economic pressure, degree of impact on life, route of HBV infection, history of oral antivirals, duration of oral antivirals (years), current treatment regimen, duration of Peg‐INF‐*α* therapy (weeks), adverse reactions, and so on.

#### 2.2.2. All Aspects of Health Literacy Scale (AAHLS)

The AAHLS comprises 11 items across 3 dimensions: corresponding to skills in using written health information, communicating with healthcare providers, and the ability to evaluate and apply health information [18]. It is rated on a 4‐point Likert scale from 1 to 3. A higher total score indicates a higher level of health literacy. The Cronbach′s alpha coefficient for the Chinese version of the AAHLS was 0.811, and the retest reliability was 0.853.

#### 2.2.3. Perceived Social Support Scale (PSSS)

The PSSS is a self‐report assessment tool consisting of 12 items designed to assess perceived social support from three sources: family, friends, and significant others [19]. Respondents were asked to report their level of agreement with the items on a 7‐point Likert scale. The aggregate of the scores for each item constitutes the overall score. The higher the score, the higher the level of social support perceived by the individual.

#### 2.2.4. The Control Preferences Scale (CPS)

The CPS scale was used in this study to evaluate the extent of patient involvement in real medical decision‐making. Participants were required to select one of five options that best described their decision‐making role, according to a comprehensive description. Subsequently, participants were classified into three decision‐making categories: active, cooperative, and passive [20].

#### 2.2.5. The Decisional Conflict Scale (DCS)

The DCS comprises 16 self‐administered items with three dimensions (information and values, decision support and decision effectiveness, and decision uncertainty). It is aimed at evaluating the level of patients′ decision conflict in the treatment decision‐making process. The scale is rated on a 5‐point Likert scale from 0 (*strongly agree*) to 4 (*strongly disagree*). A higher score on the scale indicates a greater level of decision conflict in the patient. The score is calculated by dividing the total score for the 16 questions by 16 and then multiplying by 25. The total score ranges from 0 to 100. A score greater than 25 indicates a decision dilemma problem, and a score greater than 37.5 indicates decision delay [21].

#### 2.2.6. The Decision Regret Scale (DRS)

The DRS comprises 5 items, scored on a scale from 1 (*strongly agree*) to 5 (*strongly disagree*), with Items 2 and 4 being reverse‐scored. The total score is then converted into a percentage. Higher scores on the scale indicate greater levels of decision regret, with a threshold of 25 points to differentiate between patients with high and low regret [22]. A score of 0 indicates *no decision regret*, 1–25 suggests *mild regret*, and scores above 25 indicate *moderate to severe regret* [23].

## 3. Statistical Analysis

Count data in the general information were described using frequencies and percentages. Measurement data was expressed as mean ± standard deviation for those that conformed to normal distribution and median for those that did not. Independent samples *t*‐test or ANOVA was used for between‐group analysis of normal data, and multiple independent samples Kruskal–Wallis *H*‐test was used for between‐group analysis of nonnormal data. Correlations between continuous variables were tested using Pearson or Spearman correlation. Factors influencing decision regret were analyzed using multiple linear regression. This study employed the stepwise regression method to select independent variables and used the variance inflation factor (VIF) to test for multicollinearity among the model′s independent variables. A VIF threshold of less than 5 was chosen as the acceptable limit. A significance level of *p* < 0.05 was considered statistically significant.

## 4. Results

Questionnaires were distributed to 184 patients with hepatitis B during the survey period, of which 155 questionnaires were recovered. Five incomplete paper questionnaires were excluded, and no questionnaires were excluded from the electronic questionnaires. A total of 150 questionnaires were finally included, resulting in an effective recovery rate of 81.52%. Since 20 patients were using interferon for the first time, they might have lacked experience and understanding of interferon treatment. Therefore, these patients were excluded from the study. Ultimately, data from 130 patients were included in the analysis. The participants′ ages ranged from 24 to 69 years, with a mean age of 41.42 ± 8.27 years, and 70% were male. Of these, 94 patients were from urban areas, and 36 were from rural areas. Only 27 (20.77%) knew the route of infection, and 88 (67.69%) had a history of treatment with oral antivirals. The duration of interferon use varied from 1 to 72 weeks, with a median of 25.5. Among the participants, 114 had experienced varying degrees of adverse effects, and 22 had discontinued treatment due to adverse effects.

In this study, the decision regret scores of hepatitis B patients ranged from 0 to 55 points, with an average score of 27.42 ± 16.35. Among the participants, 18 (13.85%) had no decision regret, 45 (34.60%) experienced mild regret with 1–25 points, and 67 (51.50%) reported moderate to severe regret with ≥ 26 points. Decision conflict scores varied from 0 to 100, with a median of 17.18. Additionally, 46 patients (35.38%) faced decision dilemmas, with 19 being decision delays.

Univariate analysis revealed that the factors influencing decisional regret among hepatitis B patients undergoing interferon therapy included travel time from home to hospital, treatment interruption due to adverse effects, degree of impact on life, and decision‐making roles (*p* < 0.05) (refer to Table 1).

**Table 1:** Analysis of factors influencing decisional regret in patients with hepatitis B undergoing interferon therapy.

	Number	Decisional regret	Statistical value	*p*
Gender			0.286	0.776
Male	91	27.69 ± 15.92		
Female	37	26.79 ± 17.53		
Residence			−0.325	0.746
Urban area	94	27.71 ± 16.69		
Rural area	36	26.67 ± 15.63		
Marital status			−0.205	0.838
Unmarried	13	26.54 ± 17.37		
Married	117	27.52 ± 16.32		
Age (years)			−0.321	0.749
18–44	86	27.09 ± 16.58		
≥ 45	44	28.07 ± 16.07		
Do you have any children?			0.273	0.785
No	115	27.56 ± 16.33		
Yes	15	26.33 ± 17.06		
Educational level			0.549	0.579
Junior high school or below	23	27.83 ± 17.57		
Senior high school	26	24.42 ± 17.05		
College or above	81	28.27 ± 16.35		
Employment status			4.607	0.203
Unemployed	14	25.0		
Part‐time job	11	30.0		
Full‐time job	100	30.0		
Retirement (from work)	5	17.5		
Economic pressure			1.306	0.275
None	14	26.07 ± 18.10		
Mild	28	22.50 ± 14.88		
Moderate	50	28.50 ± 15.85		
Severe	38	30.13 ± 17.18		
Travel time from home to hospital (h)			6.013	0.03
< 1	47	21.06 ± 15.98		
1–2	62	31.21 ± 15.35		
≥ 2	21	30.48 ± 16.42		
Is the route of infection clear?			0.071	0.943
No	103	27.47 ± 16.63		
Yes	27	27.22 ± 15.58		
Previous use of oral antivirals			−1.460	0.147
No	42	24.40 ± 14.15		
Yes	88	28.86 ± 17.20		
Duration of oral antivirals (year)			1.353	0.260
0	42	24.40 ± 14.15		
1–5	49	26.84 ± 17.61		
6–10	12	29.58 ± 16.16		
> 10	27	32.22 ± 16.95		
Treatment regimens			−0.687	0.494
Only Peg‐INF‐*α*	25	25.40 ± 14.64		
Peg‐INF‐*α* combination with NAs	105	27.90 ± 16.77		
Duration of Peg‐INF‐*α* therapy (weeks)			2.992	0.054
1–24	64	24.14 ± 15.57		
25–48	46	31.74 ± 16.94		
49–96	20	28.00 ± 15.84		
Have there been any adverse reactions?			−1.206	0.230
No	16	22.81 ± 17.22		
Yes	114	28.07 ± 16.20		
Was treatment interrupted due to adverse effects?			−2.277	0.024
No	108	25.97 ± 16.35		
Yes	22	34.54 ± 14.71		
Degree of impact on life			4.476	0.005
None	7	19.28 ± 12.05		
Mild	27	19.44 ± 15.77		
Moderate	66	28.94 ± 15.77		
Severe	30	33.17 ± 16.27		
Decision‐making roles			5.666	0.004
Active	23	31.95 ± 13.38		
Cooperative	69	23.04 ± 16.25		
Passive	38	32.63 ± 16.36		

Pearson correlation and Spearman correlation tests indicated strong associations between social support, health literacy, decision conflict, and decisional regret (Table 2).

**Table 2:** Factors related to decisional regret.

	Social support	Health literacy	Decision conflict	Decisional regret
Social support	1	0.194 (*p* = 0.027)	−0.213 (*p* = 0.015)	−0.181 (*p* = 0.039)
Health literacy	0.194 (0.027)	1	−0.347 (*p* < 0.001)	−0.230 (*p* = 0.009)
Decision conflict	−0.213 (0.015)	−0.347 (*p* < 0.001)	1	0.458 (*p* < 0.001)
Decisional regret	−0.181 (0.039)	−0.230 (*p* = 0.009)	0.458 (*p* < 0.001)	1

Multiple linear regression analyses were conducted with decision regret as the dependent variable. The variables that showed statistically significant results in multifactor analysis and had significant correlation coefficients were used as independent variables. Categorical variables were transformed into dummy variables. Mild impact on life, active decision‐making role, passive decision‐making role, and decision conflict collectively accounted for 29.6% of the total variance in decisional regret (*F* = 14.585, *p* < 0.001) (Table 3). Based on the *R* ^2^ value from our study, with a significance level of *α* = 0.05 and 11 independent variables (including dummy variables), we conducted a post hoc power analysis using G∗Power software. The resulting power value was 0.99, indicating that the sample size used for the regression analysis was sufficient for reliable statistical inference.

**Table 3:** Linear regression analysis of decisional regret.

Variables	*B*	*t*	*p*	95% CI	VIF
Constant	—	8.142	< 0.001	13.554–22.259	—
Active decision‐making roles	0.165	2.119	0.036	0.464–13.597	1.107
Passive decision‐making role	0.288	3.643	< 0.001	4.707–15.903	1.143
Degree of impact on life (mild)	−0.217	−2.814	0.006	−14.863 to −2.589	1.092
Decision conflict	0.401	5.319	< 0.001	0.228–0.497	1.041

## 5. Discussion

Peg‐INF‐*α* offers several advantages such as a shorter course of treatment, lower resistance rate, and higher HBsAg clearance rate compared with NAs. However, patients may experience various adverse symptoms during its use. In this study, we investigated the current situation of decisional regret in patients with hepatitis B undergoing Peg‐INF‐*α* therapy and analyzed its influencing factors, which has received little attention in previous studies. Our results showed that the decision regret score of hepatitis B patients was 27.42 ± 16.35, which was higher than the national norm score [22]. For Peg‐INF‐*α* therapy, only 13.85% of patients had no regrets at all about the decision, and the proportion of patients with moderate to severe regrets reached 51.5%, a phenomenon that deserves our attention for the high degree of decision‐making regrets in hepatitis B patients undergoing Peg‐INF‐*α* therapy.

The results of multivariate and regression analysis in this study revealed that mild impact on life, active decision‐making role, passive decision‐making role, and decision conflict were the influencing factors of decisional regret in hepatitis B patients. These factors collectively accounted for 26.4% of the total variation. Our research indicates that patients whose daily life is less influenced by interferon experience lower levels of regret regarding their decisions. Patients receiving interferon treatment must visit the hospital weekly for injections. Among the 114 patients in our survey group, representing 87.69% of the population, there were various symptoms and differing degrees of adverse reactions to interferon. All of these factors impact the patients′ daily lives.

Our research indicated that both active decision‐making (making decisions independently) and passive decision‐making (having decisions made by doctors) were associated with higher levels of decision regret. Regression analysis also showed that these two factors were significant predictors of decision regret in patients with hepatitis B. One survey showed that nearly half of patients with hepatitis B are not currently involved in treatment decisions, and 84% want to be involved in hepatitis B management and treatment choices [24]. In our survey, only 53.07% of the patients assumed collaborative roles in decision‐making. Patients who made collaborative decisions with their physicians had lower levels of regret, which is consistent with previous findings in other groups [25]. Shared decision‐making is an approach that advocates and promotes patient‐centered healthcare, helping patients choose treatment options that best fit their preferences, values, beliefs, emotional states, and perceived capabilities [26]. The process of shared decision‐making allows patients to be more aware of the benefits and risks of each option, to have a clearer understanding of the benefits and risks, and to feel confident that they will receive adequate support and advice in making their choices [27].

Another important point to consider is the status of decision conflict in hepatitis B patients. Decision conflict is a significant, subjective, and comprehensive issue characterized by uncertainty among individuals regarding the decisions they need to make. This uncertainty is reflected in their lack of clarity about alternatives, hesitation in weighing potential gains and losses when making decisions [28]. Decision conflict, as a risk factor associated with the decision‐making process, is closely linked to decision regret, which is in line with previous research findings [29]. In this study, 46 patients (35.38%) had a decision conflict score greater than 25, indicating uncertainty about the decision and the presence of a decision dilemma. Research has shown that over 80% of hepatitis B patients are treated with oral NAs, and many patients struggle to grasp the specific implications of functional cure in clinical practice. The need for long‐term treatment and the inability to achieve HBsAg loss are the primary reasons for their dissatisfaction with the treatment [30]. Peg‐INF‐*α* can partially address the limitations of conventional treatment, but its convenience and safety are relatively poor. The various adverse reactions experienced by some patients during treatment, leading to treatment interruptions, create uncertainty among patients about their decisions, such as whether another option could result in a better outcome [31]. Healthcare professionals who engage patients in shared decision‐making and provide appropriate decision aids can enhance patient knowledge, reduce decision conflict, and assist patients in making well‐informed choices aligned with their values [32, 33].

There are some potential limitations in this study. This study was a single‐center study, which has implications for the representativeness of the sample and the generalizability of the results. Second, surveys in which patients were asked to report retrospectively on their decision‐making experiences may be subject to recall bias. Additionally, this study was a cross‐sectional study and did not include patients who had completed Peg‐INF‐*α* therapy, which could not fully reflect the characteristics of decision regret in different periods. In the future, a multicenter prospective study, combined with a qualitative study, could be conducted to investigate changes in the trajectory of decision regret among hepatitis B patients regarding Peg‐INF‐*α* therapy in order to gain insight into the characteristics of their decision‐making process. This study represents our initial investigation into the decision regret experienced by hepatitis B patients undergoing interferon treatment. Although our regression model accounts for 29.6% of the variance, many variables remain unexplained and warrant further investigation in future research.

In conclusion, we found that 51.5% of hepatitis B patients treated with Peg‐INF‐*α* experienced moderate to severe decisional regret. Health professionals ought to recognize this phenomenon and provide appropriate assistance to patients. Factors such mild impact on life, active decision‐making role, passive decision‐making role, and decision conflict were identified as the main influencing factors in this study. Future research should confirm additional factors and explore the trajectory of decisional regret. Healthcare professionals should offer shared decision‐making conditions and decision support for patients.

## Funding

No funding was received for this manuscript.

## Conflicts of Interest

The authors declare no conflicts of interest.

## Data Availability

The datasets generated during and/or analyzed during the current study are available from the corresponding author on reasonable request.
